# Close association between lifestyle and circulating FGF21 levels: A systematic review and meta-analysis

**DOI:** 10.3389/fendo.2022.984828

**Published:** 2022-08-25

**Authors:** Zonghao Qian, Yucong Zhang, Ni Yang, Hao Nie, Zhen Yang, Pengcheng Luo, Xiuxian Wei, Yuqi Guan, Yi Huang, Jinhua Yan, Lei Ruan, Cuntai Zhang, Le Zhang

**Affiliations:** ^1^ Institute of Gerontology, Department of Geriatrics, Tongji Hospital, Tongji Medical College, Huazhong University of Science and Technology, Wuhan, China; ^2^ Gerontology Center of Hubei Province, Wuhan, China

**Keywords:** circulating FGF21, lifestyle, biomarker, diet, exercise

## Abstract

**Background:**

The impact of lifestyle factors on circulating fibroblast growth factor 21 (cFGF21) remains unclear. We conducted this systematic review and meta-analysis to evaluate the association between lifestyle factors and cFGF21 levels.

**Methods:**

We included studies that evaluated the effects of different lifestyles on cFGF21 concentration in adults, which included smoking, exercise, diets, alcohol consumption and weight loss. Random effects models or fixed effects models were used for meta-analysis to calculate the standardized mean difference (SMD) and 95% confidence interval according to the heterogeneity among studies. Study quality was assessed using the Newcastle–Ottawa Scale for cohort studies, the Joanna Briggs Institution Checklist for cross-sectional studies, and the PEDro scale for experimental studies.

**Results:**

A total of 50 studies with 1438 individuals were included. Overall, smoking, a hypercaloric carbohydrate-rich diet, a hypercaloric fat-rich diet, amino acid or protein restriction, excessive fructose intake and alcohol consumption significantly upregulated cFGF21 levels (p<0.05), whereas fish oil intake and calorie restriction with sufficient protein intake significantly decreased cFGF21 (p<0.05). Compared to the preexercise cFGF21 level, the cFGF21 level significantly increased within 3 hours postexercise (p<0.0001), while it significantly decreased in the blood sampled >6 h postexercise (p=0.01). Moreover, higher exercise intensity resulted in higher upregulation of cFGF21 at 1-hour post exercise (p=0.0006).

**Conclusion:**

FGF21 could serve as a potential biomarker for the assessment of different lifestyle interventions. When it is used for this purpose, a standard study protocol needs to be established, especially taking into consideration the intervention types and the sampling time post-intervention.

**Systematic Review Registration:**

https://www.crd.york.ac.uk/prospero/display_record.php?ID=CRD42021254758, identifier CRD42021254758.

## Introduction

Lifestyle is an important factor affecting individual health, which has attracted increasing attention from medical researchers. According to the data of the World Health Organization, 60% of individual health-related factors are associated with lifestyle (1). Thus, early lifestyle interventions could be highly important and effective to promote health, prevent diseases and alleviate unhealthy lifestyle-caused medical and social burdens.

Fibroblast growth factor 21 (FGF21) was first discovered by Nishimura et al. from mouse embryos in 2000 (2). In humans, FGF21 is expressed in many tissues including liver, adipose tissue, pancreas, muscle, and central nervous system. However, FGF21 is most abundantly expressed in the liver (3). As FGF21 lacks the canonical heparan-binding domain that defines the nonendocrine FGFs, it could escape from the extracellular matrix into the blood and act as an endocrine factor (4). Circulating FGF21 (cFGF21) binds to the receptor complex on the cell surface, which consists of coreceptor protein β-klotho (KLB) and FGF receptor (FGFR), especially FGFR1c, to activate FGFR signaling and regulate a variety of processes including glucose, lipid and energy metabolism, macronutrient preference and anti-inflammatory process (4–7). Therefore, long-acting analogs of FGF21 or FGF21 receptor agonists have been created to treat diabetes. The elevation of FGF21 by long-acting FGF21 analogs shows significant improvements in dyslipidemia and hepatic fat fractions in nonalcoholic steatohepatitis patients (7, 8).

cFGF21 levels have been shown to be elevated in subjects with metabolic syndrome, nonalcoholic fatty liver, hyperlipidemia, and cardiovascular diseases (9, 10). Thus, cFGF21 is regarded as a useful biomarker of metabolic disorders. In this study, lifestyles were referred to daily behaviors that are known to affect health, including smoking, alcohol consumption, diet, weight loss and exercise. Lifestyles play a major role in affecting metabolic syndrome, suggesting a possible link between cFGF21 and lifestyles. In recent years, the number of studies investigating associations between cFGF21 and different lifestyles has grown, but the results remain elusive and controversial. Therefore, this systematic review and meta-analysis was performed to summarize the findings of previous studies and to evaluate the association between cFGF21 and different lifestyles including smoking, alcohol consumption, diet, weight loss and exercise.

## Methods

### Study design

This work was executed in accordance with the Preferred Reporting Items for Systemic Reviews and Meta-analysis (PRISMA) guidelines (11). It was also registered in the International Prospective Register of Systematic Reviews (PROSPERO) before screening studies for inclusion (ID : CRD42021254758) (https://www.crd.york.ac.uk/prospero/display_record.php?ID=CRD42021254758).

### Literature search

We conducted a systematic literature search by searching PubMed, Web of Science, EMBASE and Cochrane Library in June 2021. Studies that assessed the association between serum FGF21 concentration and different lifestyles were identified. The following terms and their combinations were employed: “fibroblast growth factor 21”, “FGF21”, “lifestyle”, “smoking”, “drinking”, “alcohol drinking”, “ethanol”, “exercise”, “physical activity”, “weight loss”, “diet”, “fasting”, “sleep”, “sedentary”. The same medical subject headings (MeSH) and keywords in various combinations were used in the mentioned electronic databases. The detail search strategy in PubMed was fully described in the Supplementary Method 1.

### Selection criteria

The inclusion criteria were as follows (1): Studies assessed the association between serum or plasma FGF21 concentrations and lifestyles, including smoking, physical activity, diets, alcohol drinking and weight loss (2); Studies reported the outcomes as the mean with standard deviation or median with quartile of serum FGF21 concentration in patients with or without different lifestyles; and (3) Adult clinical studies published in English.

The exclusion criteria were as follows: (1) reviews, letters, meeting abstracts, case reports, comments and editorials; (2) duplicate studies with overlapping data; (3) studies reporting invalid data; and (4) studies on pregnant women or children.

According to the selection criteria, the initial screening of studies was based on the titles and abstracts. Then, the full texts of the potential studies were assessed. An additional manual search of references from identified studies was also performed. All studies were independently screened by two reviewers (QZH and ZYC). A third researcher (YN) was consulted to resolve disagreements.

### Data extraction and quality assessment

Two reviewers independently extracted data from the included studies. Basic information and patient baseline characteristics of all studies were extracted. To assess the association between cFGF21 and lifestyle factors, the following data were extracted: mean with standard deviation or median with quartile of cFGF21 concentration. In studies with different groups of participants, such as male and female participants, groups were extracted separately. In addition, we generally chose to extract cFGF21 level data with the longest interval period following lifestyle intervention in each study. For example, for the studies that provided both 1- and 2-day sampling points after lifestyle intervention, only the data at the 2-day sampling point were used. However, for studies studying the relationships between exercise and cFGF21, when the cFGF21 level was measured within 3 hours, we chose data of the maximum or the minimum to best reflect the temporary effect of exercise, while after 3 hours, we also extracted cFGF21 level data with the longest interval period following exercise. If there was any missing information about the eligible study, we contacted the corresponding authors and the first authors. The authors of eight included studies provided the mean ± SD or raw data (**Tables 1**–**5**, labeled with *). However, eleven studies did not provide specific data, and we extracted the data from image figures using WebPlotDigitizer (**Tables 1**–**5**, labeled with ^#^) (https://apps.automeris.io/wpd/index.zh_CN.html).

**Table 1:** Baseline characteristics of studies reported cFGF21 concentration in subjects with or without smoking.

References	Region	BMI (kg/m^2^)	Exp group (n)	Con group (n)	Male (%)	Age (years)		cFGF21 (pg/ml)	
						Exp	Con	Exp	Con
Kamizono et al., 2018 (12)	Japan		21	21				230±277	181±131
Nakanishi et al., 2015 (13)	Japan	Exp: 23.9±3.0 Con: 23.1±2.7	40	40	100	46.1±5.0	46.1±5.2	283±194	198±126
Nakanishi et al., 2018a (14) (male)	Japan	Exp: 23.4±2.7 Con: 23.2±2.5	45	45	100	47.6±6.5	47.7±6.7	280±196	198±125
Nakanishi et al., 2018b (14) (female)	Japan	Exp: 20.8±2.6 Con: 20.8±2.1	70	70	0	47.1±6.3	47.1±6.4	140±90	108±73

cFGF21, circulating fibroblast growth factor 21; Con, Control; Exp, Experimental.

**Table 2:** Baseline characteristics of studies reported cFGF21 concentration in subjects with diet intervention.

References	Region	Weight (kg)	BMI (kg/m^2^)	Description of intervention	Exp group (n)	Con group (n)	Male (%)	Age (years)	cFGF21 (pg/ml)	
									Exp	Con
Lundsgaard et al 2017 (15)	Denmark			diet enriched in carbohydrates 3 days	8	9	100	23±3	329±297	39±27
* Heilbronn et al 2013 (16)	Australia	75.3±1.9	25.6±0.6	28 days of high fat overfeeding (+1250 kCal/day, 45% fat, 15% protein, 40% carbohydrate )	35	35	50	37.6±8.8	73.9±50.9	65.6±52.4
Vienberg et al 2012a (17) (low birth weight)	Denmark			5 days of high-fat overfeeding	20	20	100		306.8±178.4	138.9±148
Vienberg et al 2012b (17) (normal birth weight)	Denmark			5 days of high-fat overfeeding	26	26	100		254.8±115.2	78.5±70.9
* Willis et al 2020 (18)	UK	76.8±3.7	24.1±1.5	7-d hyperenergetic, high-fat diet	12	12	100	24.3±4.2	85±13	67±17
Fontana et al 2016 (19)	USA	101.5±18.8	30.7±5.4	7%-9% protein-restricted	19	19	100	52.5±6.8	260.3±172.7	131.6±101.6
Gosby et al 2016 (20)	Australia		21.8±0.4	Exp: 10% protein diet 5days Con: 25% protein diet, 5days	26	26	34.6	24±1	300±48	48±48
Hollstein et al 2019 (21)	USA	62.3±3.1	20.5±1.6	low-protein overfeeding diet, 2% protein, 68% carbohydrate, and 30% fat	7	7	100	31±12	5351±1836	148±119
* Laeger et al 2014 (22)	USA	69.1±11.6	25.1±3.0	overfed with diets containing 5% protein	8	8	62.5	22.9±2.8	802.5±1198.2	450.3±758.9
Olsen et al 2020 (23)	Norway	87.4±11.7	29.9±3.4	Dietary restriction of methionine and cysteine 7 days, methionine/cysteine low group	7	7	0	31.1±5.9	202.2±120.2	134.0±100.5
^#^ Dushay et al 2015a (24) (lean group)	USA		25±3.2	oral ingestion of 75g of fructose, measured after 240 minutes	10	10	70	29±6.3	410.8±168.2	127±56.9
^#^ Dushay et al 2015b (24) (metabolic syndrome group)	USA		32±3.2	oral ingestion of 75g of fructose, measured after 240 minutes	11	11	54.5	49±9.5	1013.8±431.2	278±199.0
Migdal et al 2018 (25)	USA		23.7±0.4	fructose drink	12	12	41.6	33.1±3.9	a: 564.1±682.4 b: 737.2±746.2 c: 679.8±719.1	a: 155.2±221 b: 176.5±211 c: 216.9±267.1
Kanbay et al 2021a (26) (Group1)	Turkey		22.9±6.3	Drink 125ml apple juice every 15 minutes (totally 500ml, after 60min)	15	15	40	25.3±1.8	24.5±15.6	16.4±17.2
Kanbay et al 2021b (26) (Group2)	Turkey		22.9±2.1	Drink 500ml apple juice within 5 minutes, after 60min	15	15	46.7	26.9±4.9	43.9±17.1	18.3±15.4
Qin et al 2015 (27) (fish oil)	China	72.9±13.5	26.4±3.9	fish oil	36	36	72.2	46.0±10.7	115±10	237±28

cFGF21, circulating fibroblast growth factor 21; Con, Control; Exp, Experimental. *: data provided by the authors; #: data extracted from image.

**Table 3:** Baseline characteristics of studies reported cFGF21 concentration in subjects with or without alcohol consumption.

References	Region	Weight (kg)	BMI (kg/m^2^)	Description of alcohol consumption	Exp group (n)	Con group (n)	Male (%)	Age (years)		cFGF21 (pg/ml)	
								Exp	Con	Exp	Con
Desai et al 2017a (28)	USA		<25	Drinking ethanol >0.4g/kg body weight every 10 min in 50 min	7	7	42.9	38.4±13.2	38.4±13.2	700±921	55±37
Desai et al 2017b (28)	USA		<25	Drinking ethanol >0.9g/kg body weight every 10 min in 50 min	7	7	42.9	38.4±13.2	38.4±13.2	2194±8618	55±37
Lanng et al 2019 (29)	Denmark	77±7.9	23±2.6	intragastric ethanol infusion	12	12	100	25±3.9	25±3.9	270±215	31±35
Søberg et al 2018 (30)	Denmark		24.5±6.1	Alcohol drinking, an average of 22.6 beers/person/day (4.4 g/kg/day of ethanol) for 3 days	3	3	100	42	42	197±29.6	105.3±20.2
Wagner-Skacel et al 2021a (31) (ALC)	Austria		27±4	Alcohol drinking	9	32	22	55.4±9.2	58.0±7.0	1059±1216	333±347
Wagner-Skacel et al 2021b (31) (NALC)	Austria		27.2±4.1	Alcohol drinking	4	30	38	57.9±9.7	58.0±7.0	490±284	296±489

ALC, alcoholic liver cirrhosis; cFGF21, circulating fibroblast growth factor 21; Con, Control; Exp, Experimental; NALC, nonalcoholic liver cirrhosis.

**Table 4:** Baseline characteristics of studies reported cFGF21 concentration in subjects with weight loss intervention.

References	Region	Weight (kg)	BMI (kg/m^2^)	Description of intervention	Exp group (n)	Con group (n)	Male (%)	Age (years)	cFGF21 (pg/ml)	
									Exp	Con
^#^ Crujeiras et al 2017a (32)(discovery cohort)	Spain	95.9±16.3	35.5±4.4	ketogenic VLCD, low in carbohydrates and lipids	20	20	40	47.2±10.2	41.8±8.5	102.6±110.8
^#^ Crujeiras et al 2017b (32) (validation cohort)	Spain	91.9±10.6	33.2±1.6	ketogenic VLCD, low in carbohydrates and lipids	28	28	41.4	45.8±10.4	33.8±16.3	59.5±54.7
Lips et al 2014 (33)	Netherland	117.2±11.4	42±3.8	VLCD, high protein (~90 kcal each, of which ~18g protein, ~2.5-5g carbohydrates, 0.5-2g fat)	12	12	0	51.0±4.8	170.0±138.6	420.0±277.1
Melhem et al 2021a (34) (responders)	UK	99.8±3.2		LCD, 624 kcal/day (43% carbohydrate, 34% protein and 19.5% fat)	12	12	66.7	52.0±2.9	263.4±82.6	510.4±76.5
Melhem et al 2021b (34) (non-responders)	UK	96.7±3.9		LCD, 624 kcal/day (43% carbohydrate, 34% protein and 19.5% fat)	17	17	41.2	59.9±2.1	552.5±119.7	511.9±69.6
Watanabe et al 2020 (35)	Italy	104.6±15.3	38.3±6	first phase lasting 45-day with a ketogenic VLCD (Carbohydrates 14%, fat 40%, protein 46%)	45	45	35.4	51.0±11.2	73.5±55.5	180.1±88.9
^#^ Xu et al 2020a (36) (high protein group)	German	139.6±21.4	44.5±3.4	LCD (1500-1600 kcal/d) diet, 30E% protein, 25-30E% fat, 35-45E% carbohydrates	7	7		49.0±23.8	119.7±85.7	183.1±104.2
^#^ Xu et al 2020b (36) (low protein group)	German	133.2±23.7	45.2±3.8	LCD (1500-1600 kcal/d) diet, 10E% protein, 25-35E% fat, 55-65E% carbohydrates	10	10		47.0±28.5	485.9±218.2	340.8±240.6
* Gomez-Ambrosi et al 2017 (37)	Spain	95±15	32.7±4.9	daily caloric deficit of 500-1000 kcal/d than the resting energy expenditure (54, 30 and 16% carbohydrates, fat, and protein)	22	22	67.9	41.8±16.4	211.8±156.9	276.5±219.1
Johansson et al 2019 (38)	Sweden	114.3±12.1	41.7±2.6	LCD carbohydrates 52%, protein 25% and fat 21%)	10	10	0	42.7±8.9	452±533	244±213
Mai et al 2011 (39)	German	97.4±17	35.3±5.5	6-month LCD (50% carbohydrates, 30% fat, 20% protein) and at least 60 minutes of physical activity per week	30	30	20	51.8±13.1	1210±274	1220±274
* Srámková et al 2016 (40)	Czech	93.5±9.5	32.7±3.7	VLCD, 800 kcal/d, 52g protein, 118g carbohydrate and 12.9g fat	17	17	0	35±28.9	311±175	196±119
Christodoulides et al 2009a (41)	UK	82.1±14.7	25.7±4.4	48-h fasting	8	8	100	26.2±2.9	155±61	143±104
Christodoulides et al 2009b (41)	UK	96.1±15.9	35.8±6	low-carbohydrate ketogenic diet (≤40 g carbohydrate/d)	7	7	100	55.4±5.4	201±134	349±193
^#^ Crujeiras et al 2017c (32)(regainers)	Spain	99.8±18.6	36.1±4.8	LCD, 40% to 55% of energy intake from carbohydrates, 30% from lipids and 15% to 30% from proteins	28	28	51.2	49.5±9.2	159.1±31.8	220.5±37.8
^#^ Crujeiras et al 2017d (32) (non-regainers)	Spain	99.8±18.6	36.1±4.8	LCD, 40% to 55% of energy intake from carbohydrates, 30% from lipids and 15% to 30% from proteins	51	51	51.2	49.5±9.2	118.2±22.0	159.1±27.3
Headland et al 2019a (42) (IER)	Australia	93.9±15.6	33.2±4.3	4200 kJ/day for women or 5500 kJ/day for men	20	20	15	52.9±12.2	600±900	500±800
Headland et al 2019b (42) (CER)	Australia	86.8±11.7	31.4±2.9	4200 kJ/day for women or 5500 kJ/day for men	23	23	26	53.7±7.7	1300±1900	1000±1400

CER, continuous energy restriction; cFGF21, circulating fibroblast growth factor 21; Con, Control; Exp, Experimental; IER, intermittent energy restriction; LCD, low calorie diet, VLCD, very low calorie diet. *: data provided by the authors; #: data extracted from image.

**Table 5:** Baseline characteristics of studies reported cFGF21 concentration in subjects with or without physical activity intervention.

References	Region	Weight (kg)	BMI (kg/m^2^)	Postexercise sampling time	Description of exercise	Exp group (n)	Con group (n)	Male (%)	Age (years)	cFGF21 (pg/ml)	
										Exp	Con
Cuevas-Ramos et al 2012 (43)	Mexico		21.4±7.0	unclear	a bout of exercise (treadmill) and repeated after 2 weeks of daily supervised exercise, heart rate above an intensity of 85% for at least 15min	60	60	0	24.0±3.7	502.7±337.2	332.5±323.4
* J̈rimäe et al 2021 (44)	Estonia	67.5±8.8		immediately after	aerobic rowing exercise, 1h, 70% of the subject’s VO_2_ max	15	15	0	18.3±1.6	168.9±68.8	147.6±65.3
^#^ Kim et al 2013a (45)	Korea	68.4±4.7	21.4±1.8	after 1h	treadmill running for 30min at 50% VO_2_ max	13	13	100	22.1±1.1	134.6±42.2	56.6±28.1
^#^ Kim et al 2013b (45)	Korea	68.4±4.7	21.4±1.8	after 1h	treadmill running for 30 min at 80% VO_2_ max	8	8	100	22.1±1.1	249.1±101.3	71.6±28.3
^#^ Morville et al 2018a (46)	Denmark	80.6±5.9	23.7±1.7	60 minutes 180 minutes	endurance exercise (1h of bicycling at 70% of VO_2_ peak)	8	8	100	24±2.8	60min: 166.0±90.5 180min: 40.6±22.1	46.0±31.1
^#^ Morville et al 2018b (46)	Denmark	80.6±5.9	23.7±1.7	60 minutes 180 minutes	resistance training, 58–59min	8	8	100	24.0±2.8	60min: 66.6±34.8 180min: 38.7±26.3	62.0±48.1
^#^ Sabaratnam et al 2018a (47) (Con)	Denmark		29.0±0.9	immediately after 60 min	acute exercise (60min cycling)	14	14	100	54.7±2.3	86.5±9.6	81.3±7.5
^#^ Sabaratnam et al 2018b (47)(with T2DM)	Denmark		29.7±1.0	immediately after 60 min	acute exercise (60min cycling)	13	13	100	55.4±2.0	243.3±30.8	209±36.6
^#^ Sargeant et al 2018a (48) (normal weight)	UK	69.8±1.5	23.4±1.6	after 0.5 h, after 6 h	60min bout of moderate-intensity treadmill exercise (60% of V̇O_2_ peak)	11	11	100	36±15	after 0.5h: 183.4±150.6 after 6h: 74.4±39.1	83±55
^#^ Sargeant et al 2018b (48) (overweight/ obese )	UK	92.3±3.4	29.2±4.5	after 0.5 h, after 6 h	60min bout of moderate-intensity treadmill exercise (60% of V̇O_2_ peak)	11	11	100	45±14	after 0.5h: 293±107 after 6h: 15±69	190±74
^#^ Slusher et al 2015a (49) (normal-weight)	USA	64.2±12.2	22±1.6	Post 1h	30min aerobic exercise at 75% of the subject’s VO_2_ max	12	12	50	23.2±2.2	189.4±88.7	116.0±63.0
^#^ Slusher et al 2015b (49) (obese)	USA	99.1±17.3	35.5±4.1	Post 1h	30min aerobic exercise at 75% of the subject’s VO_2_ max	12	12	41.7	23.2±2.2	131.0±65.8	103.8±42.3
* Taniguchi et al 2016a (50) (young participants)	Japan	69.3±7.4	21.3±1.3	post 60min postday	acute endurance exercise (cycling exercise for 30min at 70 % VO_2_ max)	7	7	100	20.8±1.1	post 60min: 235.9±171.1 postday: 120.2±61.3	200.4±140.7
* Taniguchi et al 2016b (50) (elderly participants)	Japan	64.3±7.6	23.0±2.9	post 60min postday	acute endurance exercise (cycling exercise for 30 min at 70 %VO_2_ max)	8	8	100	64.3±1.6	post 60min: 464.0±126.8 postday: 267.5±125.9	379.2±156.3
^#^ Tanimura et al 2016 (51)	Japan	66.0±6.9	22.4±2.0	immediately after	Exercise on bicycle ergometers for 60min at 75% of their VO_2_ max	19	19	100	23.7±2.3	157.9±143.0	125.5±126.6
* Willis et al 2019a (52)	UK	80.2±6.2	25.6±1.7	post 1h, post 7h	treadmill run high-intensity (70% VO_2_ peak),	10	10	100	26±2	post 1h: 194±159 post 7h: 78±50	63±47
* Willis et al 2019b (52)	UK	80.2±6.2	25.6±1.7	post 1h, post 7h	treadmill run, moderate intensity exercise (55% VO_2_ peak)	10	10	100	26±2	post 1h: 81±53 Post 7h: 78±63	82±56
Lee et al 2021 (53)	Korea	63.5±9	25.1±2.5	after 12h of fasting	high-intensity circuit training, 60-80% heart rate reserve	10	10	0	21±1	237.3±302.4	270.1±257.8
Banitalebi et al 2019a (54)	Iran	77.4±12	29.3±3	48h after the last exercise session	sprint interval training 10-week	14	14	0	55.1±5.9	229.6±94.9	170.1±71
Banitalebi et al 2019b (54)	Iran	72.6±11.5	28.7±4.3	48h after the last exercise session	combined aerobic and resistance training 10-week	14	14	0	55.1±5.9	204.7±111.4	176.3±94.2
Besse-Patin et al 2014 (55)	France	102.6±7.4	32.7±2.5	after 48-72h	8-week endurance training (cycling and running), 35-85% VO_2_ max	11	11	100	35.4±1.5	143.8±33	123.1±21.5
Motahari Rad et al 2020a (56)	Iran	86.7±7.6	29.6±1.7	after 12h of fasting	aerobic-resistance training 12 weeks	17	17	100	43.9±2.5	441.7±110.0	460.5±136.7
Motahari Rad et al 2020b (56)	Iran	89.9±7.2	29.5±1.3	after 12h of fasting	resistance-aerobic training 12 weeks	17	17	100	44.0±2.6	449.0±98.8	456.6±85.7
^#^ Pérez-López et al 2021a (57)	Spain	86.4±11.5		after 8-10h of fasting	12-week concurrent training	13	13	0	58.7±2.9	195.4±81.1	205.7±51.2
^#^ Pérez-López et al 2021b (57)(postmenopausal women)	Spain	84.7±9.1		after 8-10h of fasting	12-week endurance training	10	10	0	56.7±3.7	144.3±49.5	188.6±62.7
^#^ Pérez-López et al 2021c (57) (premenopausal women)	Spain	93.2±7.0		after 8-10h of fasting	12-week endurance training	12	12	0	43.1±2.8	137.6±46.0	179.5±59.3
Shabkhiz et al 2021a (58) (without T2DM)	Iran	73.4±13.3	27.6±3.6	48h, following an overnight fast (10 hours)	12-week resistance training, 3 sets of 10 repetitions at 70% of their 1RM	12	12	100	72.1±5.3	253.2±116.1	336.1±95.4
Shabkhiz et al 2021b (58) (with T2DM)	Iran	74.6±11.6	25.41±3.5	48h, following an overnight fast (10 hours)	12-week resistance training, 3 sets of 10 repetitions at 70% of their 1 RM	10	10	100	72.5±6.0	324.1±107.7	395.9±93.0
Takahashi et al 2020 (59) (with NAFLD)	Japan	73.5±11.6	28.8±3.9	under overnight fasting	12-week resistance exercise, 3 sets of 10 repetitions at 70% of their 1 RM	23	23	34.8	55.5±12.2	142.9±105.9	184.6±113.3
Taniguchi et al 2016c (60)	Japan	64.0±8.9	23.1±2.6	after an overnight fast	5-week endurance exercise, 60%-75% of their VO_2_ max	27	27	100	69.6±4.2	218.5±94.2	248.1±88.5
^#^ Yang et al 2011 (61)	Korea	68.4±8.2	27.6±2.4	after an 8-h fast	A 3-month combined aerobic and resistance exercise program, 40-75% of the maximal heart rate	40	40	0	45.3±9.5	102.6±117.8	230.2±135.9

cFGF21, circulating fibroblast growth factor 21; Con, Control; Exp, Experimental; RM, repetition maximum. *: data provided by the authors; #: data extracted from image.

Quality assessment was independently performed by two reviewers (NH and YZ). Discrepancies were resolved by discussion with a third reviewer (LPC). The quality of cohort studies was assessed by using the Newcastle–Ottawa Quality Assessment Scale (NOS) (62), as reported in **Supplementary Table 1**. Studies scored > 5 were considered to be high-quality. The quality of the cross-sectional studies was assessed by using the Joanna Briggs Institution (JBI) Checklist for Analytical Cross-Sectional Study (https://jbi.global/critical-appraisal-tools), as reported in **Supplementary Table 2**. A score of 4 to 6 indicated moderate quality, whereas as score of 7 or more indicated high quality (63). The methodological quality of the included experimental studies was rated using the PEDro scale (https://pedro.org.au/english/resources/pedro-scale/), as reported in **Supplementary Table 3**. Publication bias was assessed by funnel plots if the number of included cohorts was ≥10. Publication bias was considered to be significant if the funnel plot was asymmetric.

### Data analysis

This meta-analysis was performed by using RevMan 5.3 (the Nordic Cochrane Center, Copenhagen, Denmark). Heterogeneity was tested by using the Chi-squared test and I^2^ statistic. p<0.05 or I^2^ >50% indicated that the heterogeneity was significant. A fixed-effects model was used to calculate the pooled estimates if no significant heterogeneity was identified (I^2^<50%); otherwise, a random-effects model was used. The overall effects were determined by the Z-test and p<0.05 was considered as statistically significant. Subgroup analysis was conducted according to specific lifestyle.

Medians with quartiles were transformed into means with standard deviations for pooled estimates by using the webpage tool in the BOX-COX manner developed by McGrath et al. (64).

## Results

### Study selection, characteristics and quality assessment

**Figure 1:**
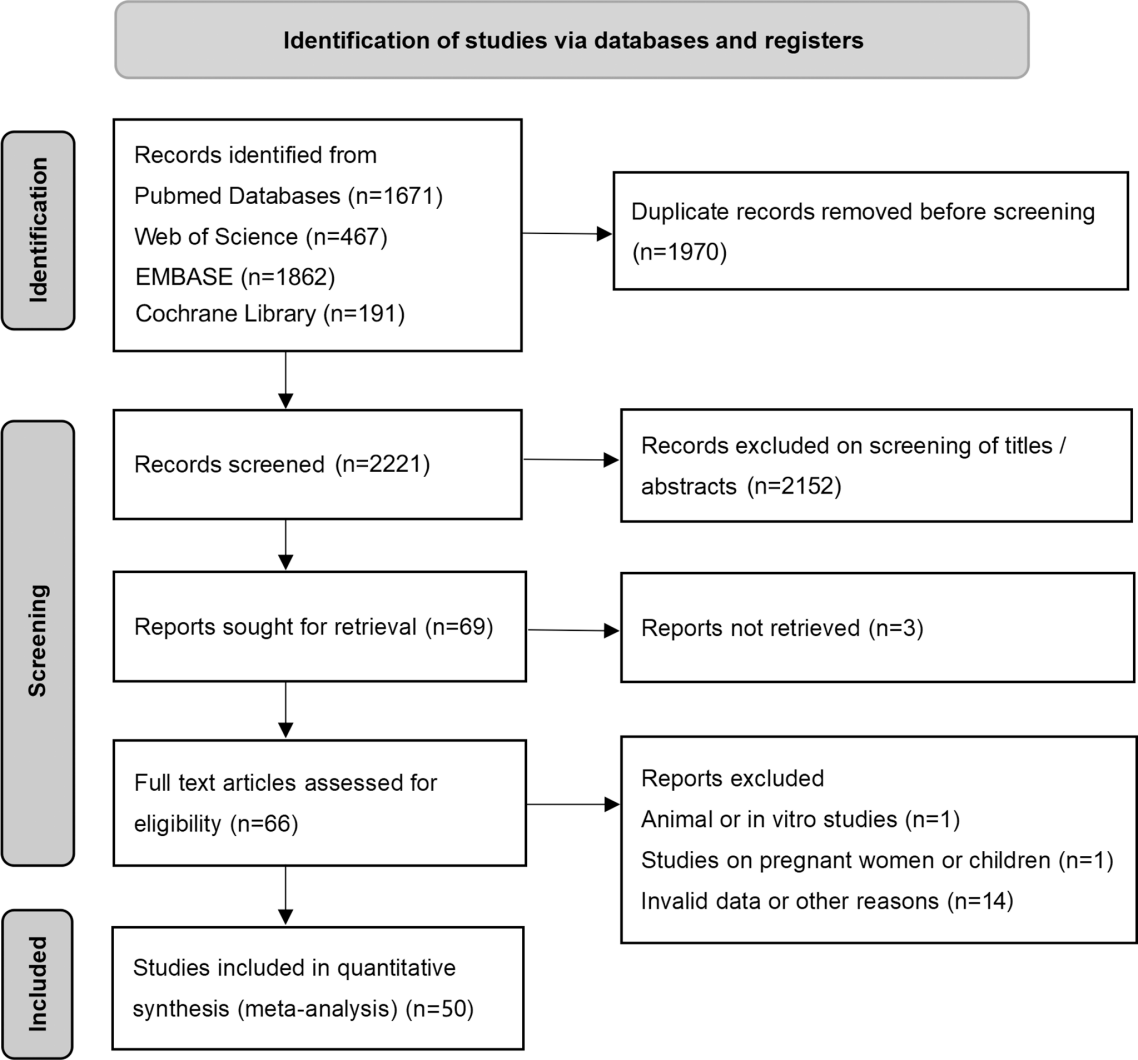
Preferred reporting items for systemic reviews and meta-analysis flow diagram of literature screening. cFGF21, circulating fibroblast growth factor 21.

The flow diagram of the search and screening process is shown in **Figure 1**. After removing duplicate articles, 2221 articles were identified in the initial database search. After screening titles and abstracts, 69 articles remained for further full-text evaluation. Finally, a total of 50 studies with 1438 individuals were included in this meta-analysis.

**Tables 1**–**5** present the characteristics of all included studies. These studies were published between 2011 and 2021. Patients were from 17 countries including Japan, USA, Austria, Australia, Iran, France, Spain, Korea, Mexico, UK, China, German, Czech, Denmark, Norway, Turkey and Netherland. According to the NOS, the cohort study was considered to be high quality (**Supplementary Tables 1**). According to the JBI checklist, all cross-sectional studies were also considered to be high quality (**Supplementary Table 2**). For the PEDro scale, a quality of study scoring less than 4 is considered ‘poor’, 4 to 5 is considered ‘fair’, and 6 to 9 is considered ‘good’ (65). Therefore, 18 studies were rated as fair quality, while 29 studies were rated as good quality (**Supplementary Table 3**).

### The association between smoking and cFGF21 concentration

**Figure 2:**
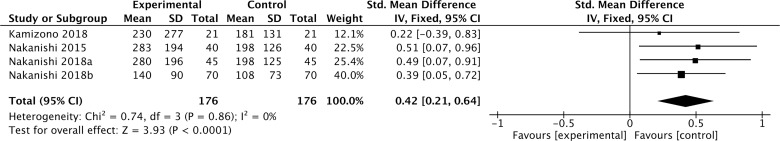
Forest plot of cFGF21 concentration in individuals with or without smoking. Fixed effect models were used as the pooling method. The mean value refers to the mean cFGF21 concentration in each group. cFGF21, circulating fibroblast growth factor 21.

Three studies containing 176 individuals were involved in the analysis of the association between smoking and cFGF21 concentration (12–14) (**Table 1**). Overall, the cFGF21 concentration was significantly higher in smokers (Experimental) than in nonsmokers (Control) (SMD=0.42 pg/ml, 95% CI: 0.21-0.64, p<0.0001) (**Figure 2**). The heterogeneity among these studies was insignificant (p=0.86).

### The association between diet and cFGF21 concentration

**Figure 3:**
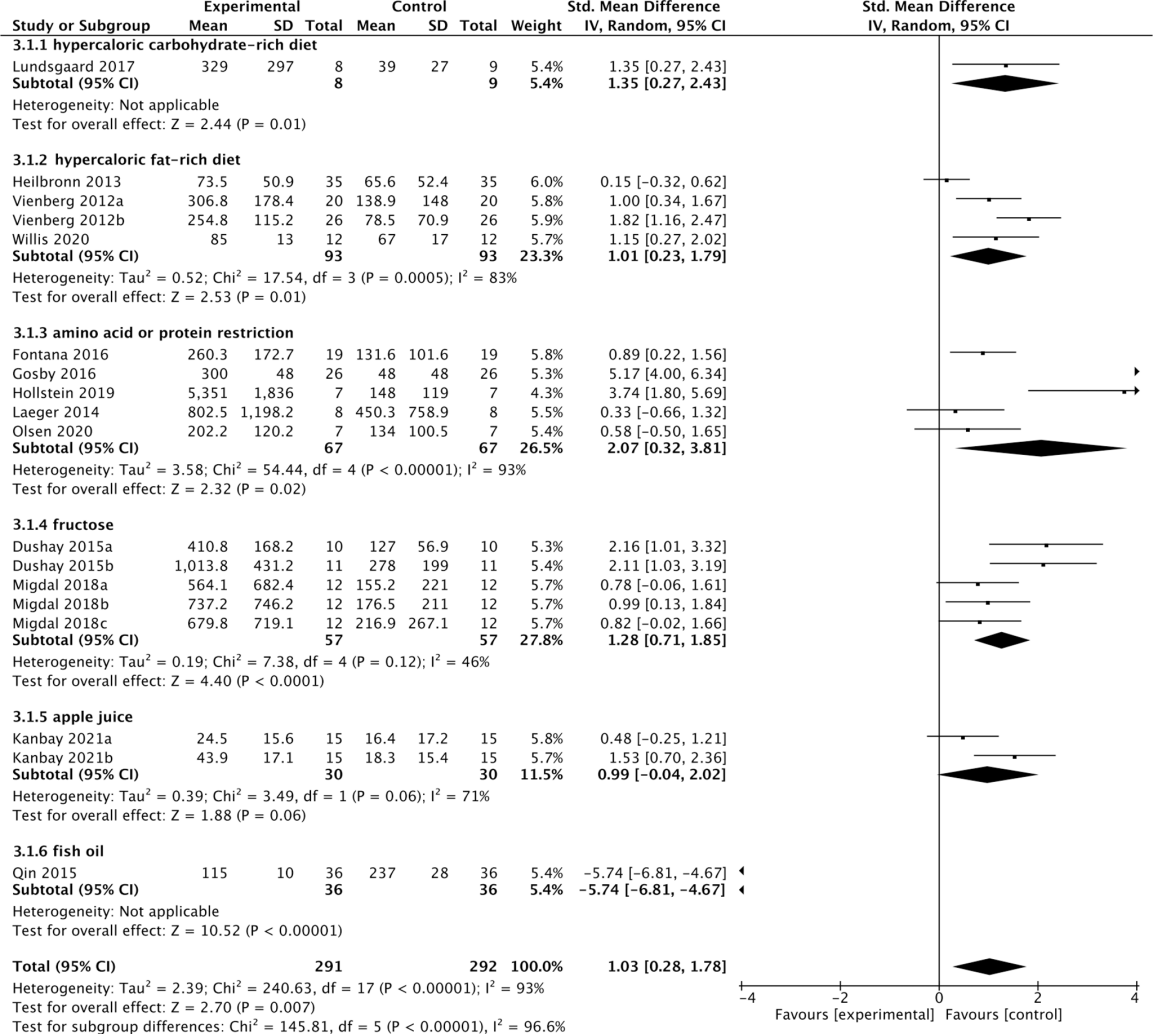
Forest plot of cFGF21 concentration in individuals before and after different diet interventions. Random effect models were used as the pooling method. The mean value refers to the mean cFGF21 concentration in each group. cFGF21, circulating fibroblast growth factor 21.

Thirteen studies containing 300 individuals were involved in the analysis of the association between diets and cFGF21 concentration (15–27) (**Table 2**). Overall, the cFGF21 concentration was significantly higher in individuals with different diets (SMD=1.03 pg/ml, 95%CI: 0.28-1.78, p=0.007) (Experimental) compared to their cFGF21 levels before indicated diet interventions (Control), largely contributed by the significant elevation of cFGF21 in individuals with a hypercaloric carbohydrate-rich diet (15) (p=0.01), hypercaloric fat-rich diet (16–18) (p=0.01), amino acid- or protein-restricted diets (19–23) (p=0.02) and fructose intake (24, 25) (p<0.0001) (**Figure 3**). While a quick and excessive drink of apple juice significantly increased the cFGF21 level, the overall effect of apple juice consumption on the cFGF21 level was insignificant (26) (p=0.06), due to a nonsignificant increase in cFGF21 after a slow drink of apple juice. Only consuming fish oil significantly downregulated the cFGF21 concentration (27) (p<0.00001) without affecting the overall effects of diets on the cFGF21 concentration (p=0.007). The heterogeneity among these studies was significant (p<0.00001). Publication bias was assessed by a funnel plot, which indicated high publication bias (**Supplementary Figure 1**).

### The association between alcohol consumption and cFGF21 concentration

**Figure 4:**
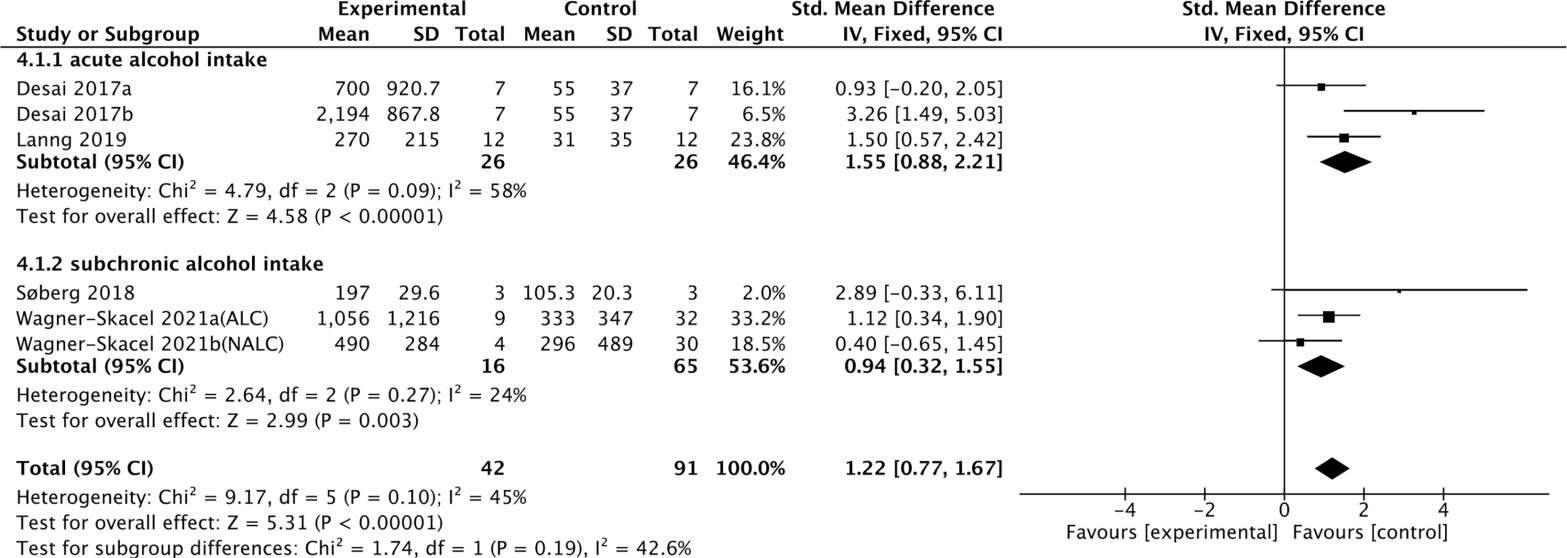
Forest plot of cFGF21 concentration in individuals before and after alcohol consumption. Fixed effect models were used as the pooling method. The mean value refers to the mean cFGF21 concentration in each group. cFGF21, circulating fibroblast growth factor 21.

Four studies containing 104 individuals were involved in the analysis of the association between alcohol consumption and cFGF21 concentration (28–31) (**Table 3**). The cFGF21 levels were significantly elevated in individuals after both acute alcohol intake (cFGF21 levels measured within 4h post alcohol consumption) (28, 29) (p<0.00001) and subchronic alcohol intake (cFGF21 levels measured in the last 12-72h post-alcohol consumption) (30, 31) (p=0.003) (Experimental) compared to their cFGF21 levels before alcohol consumption (Control), resulting in an overall elevation of cFGF21 by alcohol consumption (SMD=1.22 pg/ml, 95%CI: 0.77-1.67, p<0.00001) (**Figure 4**). In addition, the alcohol consumption-induced cFGF21 elevation was higher in patients with alcohol liver cirrhosis (ALC) than in those without (NALC) (31). The heterogeneity among these studies was insignificant (p=0.10).

### The association between calorie restriction-induced weight loss and cFGF21 concentration

**Figure 5:**
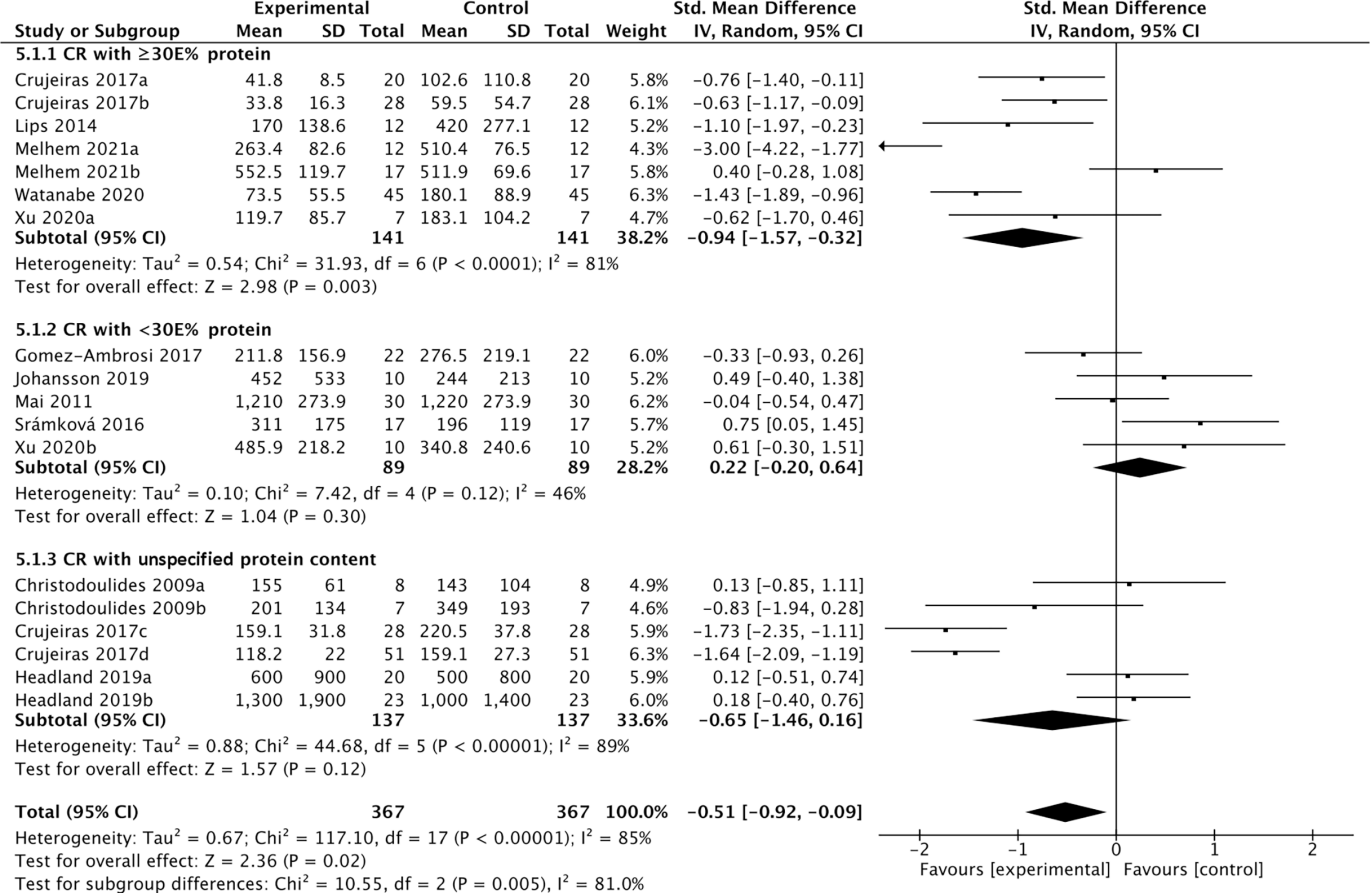
Forest plot of cFGF21 concentration in individuals before and after calorie restriction-induced weight loss. Random effect models were used as the pooling method. The mean value refers to the mean cFGF21 concentration in each group. cFGF21, circulating fibroblast growth factor 21.

Eleven studies containing 367 individuals were involved in the analysis of the association between calorie restriction (CR)-induced weight loss (CRIWL) and cFGF21 concentration (32–42) (**Table 4**). Overall, CRIWL significantly decreased the cFGF21 level (SMD=-0.51 pg/ml, 95%CI: -0.92 to -0.09, p=0.02) (**Figure 5**). Among them, cFGF21 concentrations were significantly lower in individuals who underwent hypocaloric diets with relatively high protein contents (≥30% diet calorie from protein, ≥30E% protein) than their cFGF21 levels before the diets (32–36) (p=0.003); while CR with <30E% protein (36–40) or CR with unspecified protein content (32, 41, 42) did not significantly affected the cFGF21 level (**Figure 5**, p=0.30 and p=0.12 respectively). The heterogeneity among these studies was significant (p<0.00001). Publication bias was assessed by a funnel plot, which indicated moderate publication bias (**Supplementary Figure 2**).

### The association between acute exercise and cFGF21 concentration

**Figure 6:**
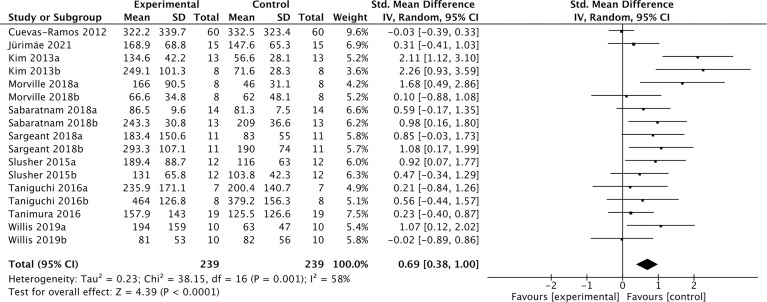
Forest plot of acute exercise effects on cFGF21 concentration sampled within 3 hours postexercise. Random effect models were used as the pooling method. The mean value refers to the mean cFGF21 concentration in each group. cFGF21, circulating fibroblast growth factor 21.

Nineteen studies containing 491 individuals were involved in the analysis of the association between physical activity and the cFGF21 concentration (43–61) (**Table 5**). Among them, 10 studies containing 239 individuals measured cFGF21 levels before (Control) and within 3 hours postexercise (Experimental) (43–52). They were then used to evaluate the effect of acute exercise on the cFGF21 level (**Figure 6**). Overall, the cFGF21 concentrations were significantly higher in the blood sampled within 3 hours postexercise than in that sampled preexercise (SMD=0.69 pg/ml, 95%CI: 0.38-1.00, p<0.0001) (**Figure 6**). The heterogeneity among these studies was significant (p=0.001). Publication bias was assessed by a funnel plot, which indicated moderate publication bias (**Supplementary Figure 3**).

### The association between exercise intensity and cFGF21 concentration

**Figure 7:**
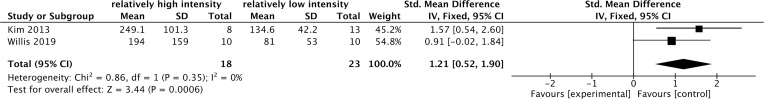
Forest plot of cFGF21 concentration in individuals with different exercise intensities. Fixed effect models were used as the pooling method. The mean value refers to the mean cFGF21 concentration in each group. cFGF21, circulating fibroblast growth factor 21.

Among the previous 10 studies related to the effects of acute exercise on cFGF21 (**Figure 6**), 2 studies containing 31 individuals were involved in the analysis of the association between exercise intensity and the cFGF21 concentration 1-hour postexercise of different intensities were compared in both studies (45, 52) (**Table 5**). Overall, the 1-hour postexercise cFGF21 concentration was significantly higher in individuals who underwent relatively higher intensity exercises than in those who underwent relatively lower intensity exercises (SMD=1.21 pg/ml, 95%CI: 0.52-1.90, p=0.0006) (**Figure 7**). The heterogeneity among these studies was insignificant (p=0.35).

### Effect of exercise after more than 6 hours of recovery on the cFGF21 level

**Figure 8:**
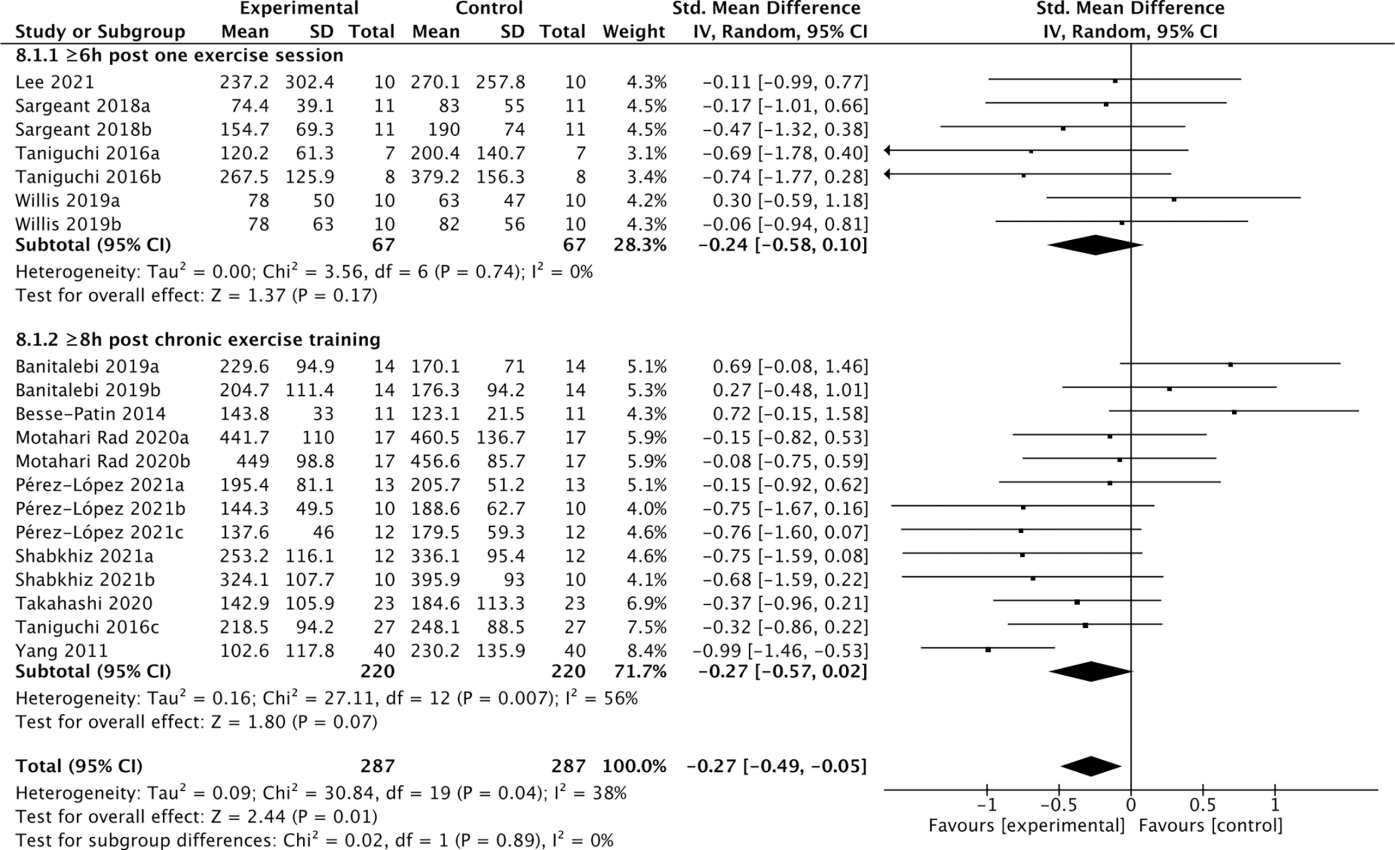
Forest plot of exercise effects on cFGF21 concentration sampled more than 6 hours postexercise. Random effect models were used as the pooling method. The mean value refers to the mean cFGF21 concentration in each group. cFGF21, circulating fibroblast growth factor 21.

**Figure 9:**
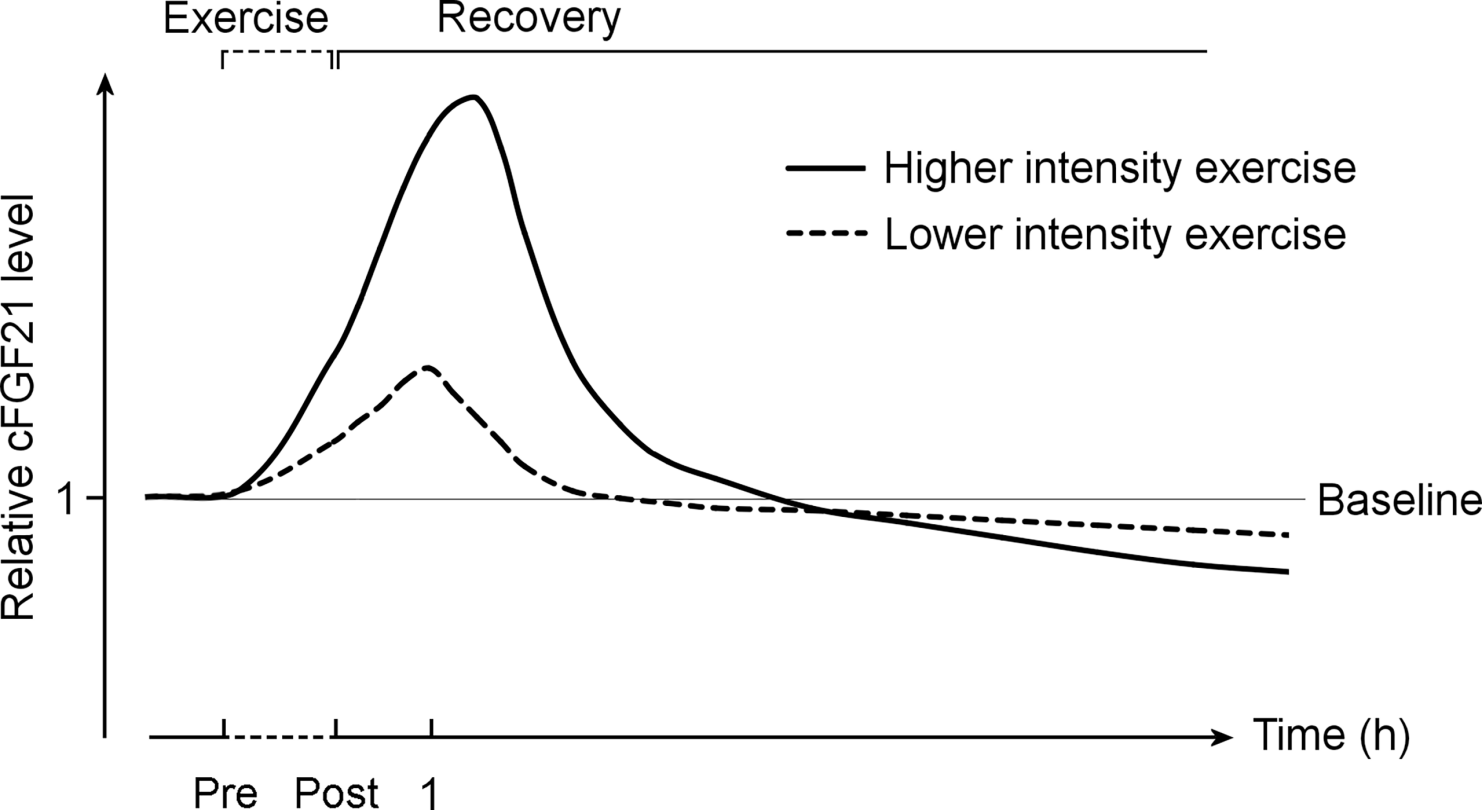
The exercise-induced cFGF21 level changes over time and with different exercise intensities. The figure includes two curves depicting changes in relative cFGF21 levels in subjects undergoing relatively higher- and lower-intensity exercises during the pre-exercise (Pre) and post-exercise (Post) periods. cFGF21, circulating fibroblast growth factor 21.

Among the 19 studies of the effects of exercise (**Table 5**), 12 studies containing 287 individuals measured cFGF21 levels in the blood sampled beyond 3 hours postexercise (48, 50, 52–61) (**Table 5**). Among these 12 studies, 4 studies (48, 50, 52, 53) measured cFGF21 concentration of individuals at least 6 hours after one exercise session, while the other 8 studies (54–61) evaluated the effects of chronic training (at least longer than 5 weeks) on the cFGF21 level at least 8 hours after the last exercise session of the whole training program. In both subgroups, the cFGF21 levels tended to decrease after exercise (Experimental) with limited significance compared to those before exercise (Control) (**Figure 9**, p=0.17 and p=0.07 respectively). Overall, the cFGF21 level was significantly lower in the blood sampled more than 6 hours postexercise (Experimental) than that of preexercise (Control) (SMD=-0.27 pg/ml, 95%CI: -0.49 to -0.05, p=0.01) (**Figure 8**). The heterogeneity among these studies was significant (p=0.04). Publication bias was assessed by a funnel plot, which indicated moderate publication bias (**Supplementary Figure 4**).

## Discussion

FGF21 has multiple metabolic regulatory effects, including stimulating hepatic oxidation of fatty acids, decreasing hepatic triglyceride accumulation, increasing insulin sensitivity and suppressing preference for alcohol and sugar intake through the central nervous system (4, 66). Even though the long-acting FGF21 analogs did not decrease plasma glucose levels in humans, they successfully decreased serum lipids, increased serum adiponectin levels, and showed several effects on weight loss (7, 8). Therefore, the elevation of cFGF21 may benefit metabolism regulation and be considered as a useful biomarker to assess the effects of metabolism interactions.

The present systematic literature review and meta-analysis showed significant impacts of different lifestyles on the cFGF21 level. The elevation of the cFGF21 level may be an excellent indicator for lifestyle intervention-induced global metabolic and cardiovascular systems and may be a protective and/or compensatory response to inflammation and stress under stressed conditions.

## Smoking and cFGF21

Smoking is the leading preventable cause of mortality and morbidity in modern society. It is well known that smoking is a pivotal risk factor for various diseases, such as cardiovascular disease and metabolic disease. The anti-inflammatory property of FGF21 has been revealed in many studies (67–69). It is known that smoking promotes inflammation by stimulating the release of proinflammatory cytokines such as interleukin-6 (70). A study demonstrated that interleukin-6 concentrations were significantly higher among smokers than never smokers (14). As is also shown in this meta-analysis, smoking significantly upregulates cFGF21 levels, suggesting that it might be a compensatory response to inflammatory stress induced by smoking.

## Hypercaloric diet and cFGF21

FGF21, initially identified as a fasting hormone, could be increased by prolonged fasting, hypercaloric fat-rich or carbohydrate-rich diets through different mechanisms (66). Prolonged fasting leads to the release of free fatty acids (FFAs) as an energy source through fatty acid oxidation, while a hypercaloric fat-rich ketogenic diet causes hepatic and plasma triglyceride accumulation. Both ways converge to increase circulating FFAs which is also a signal leading to transcriptional upregulation of FGF21, and in turn promotes FFA consumption as well as the disposal of the excessive fat load (66, 71). Hypercaloric carbohydrate-rich diets could activate the hepatic transcription factor carbohydrate response element binding protein (ChREBP), which binds directly to the FGF21 promoter and enhances the expression of FGF21 (6, 72), which in turn acts directly in the brain to suppress sugar appetite and consumption by targeting hypothalamic networks (73). Our meta-analysis is in line with these previous studies by showing significant increases in cFGF21 levels following acute hypercaloric carbohydrate-rich (15) and hypercaloric fat-rich feeding (16–18) (**Figure 3**). The limited number of studies included for the meta-analyses of these 2 subgroups could be due to limited studies for ethical reasons. In both cases, elevation of cFGF21 is thought to be a defensive response for maintenance of glucose and lipid homeostasis and regulation of macronutrient preference (6, 66).

## Protein or amino acid restriction and cFGF21

FGF21 as an endocrine signal of protein restriction, could be highly upregulated by a low-protein diet and in turn shifts macronutrient preference to increase protein appetite and intake through neural circuits in a β-klotho-dependent manner (6, 22). This was confirmed by this meta-analysis, which showed that both decreased consumption of amino acids (AAs), such as methionine/cysteine (23) or branched-chain amino acids (BCAAs: leucine/isoleucine/valine) (19) and dietary protein restriction (20–22) significantly increased the cFGF21 level of individuals (**Figure 3**). Classic endoplasmic reticulum stress response pathways (GCN2, PERK, ATF4, etc.) were reported to mediate the restriction-induced cFGF21 elevation, with the involvement of amino acid response elements (AAREs) in the FGF21 promoter (6). Moreover, AA restriction is thought to contribute to lipid and fatty acid metabolism, including lipolysis and fatty acid oxidation, thus elevating cFGF21 (19, 23). It has been reported that the maximal elevation of FGF21 occurs when protein restriction is coupled with high carbohydrate intake in mice (74); however, direct evidence for such a synergistic effect still needs to be further investigated in humans.

## Fructose consumption and cFGF21

Excessive and quick fructose consumption acutely stimulates cFGF21 levels (**Figure 3**) (24, 25) even with a dose as low as 20 g fructose for lean and healthy individuals (25). Natural fruits having a high fructose-to-glucose ratio (≥2:1) include apples, pears, watermelons and mangoes (75, 76). Apple juice, fruit drinks, regular soda and high-fructose-corn-syrup were associated with a 4-5 times higher likelihood of childhood asthma *via* fructose-induced inflammation and respiratory distress, than never/seldom consumers (p=0.035/p=0.005) (76, 77). Multiple mechanisms are thought to be involved in fructose-triggered FGF21 upregulation. First, free glucose may also stimulate FGF21 expression and secretion in a hepatic ChREBPβ-dependent manner (78). Second, unabsorbed excess-free-fructose may contribute to enteral formation of immunogens, the proinflammatory advanced glycation end products, *via* reaction with partially digested dietary proteins and enhance cFGF21 levels through increased inflammation (76, 79). Third, the fructose-dietary protein interaction in the gut may also produce a temporary decrease in dietary protein levels (76), which is in turn a strong signal for FGF21 upregulation (6, 22). This could be the reason why an excessive and quick consumption of apple juice (500 mL of apple juice drunk over 5 min) stimulated much higher FGF21 responses than a slow drink of the same amount apple juice (500 mL over an hour by drinking 125 mL every 15 min) (**Figure 3**, **Table 2**). Thus, cFGF21 could serve as an exemplary biomarker for the evaluation of excessive fructose intake from foods and/or drinks.

## Alcohol consumption and cFGF21

Alcohol consumption is another preventable major cause of morbidity and mortality globally (80). Studies have reported a dose-response relationship between cFGF21 and liver fat content in nonalcoholic fatty liver disease (81). In the liver, chronic alcohol use can cause hepatic fat accumulation and inflammation that are associated with a broad spectrum of liver diseases from simple liver steatosis to liver fibrosis, cirrhosis, and cancer (82), all of which could lead to an increase in cFGF21 levels in patients with liver and metabolic disorders. Moreover, acute alcohol intake can elevate cFGF21, which could in turn cross the blood–brain barrier and suppress further alcohol intake through a specific subpopulation of β-klotho-expressing neurons in the basolateral amygdala in alcohol-preferring nonhuman primates (83). In this meta-analysis, cFGF21 levels were elevated by both acute (within 4 hours) (28, 29) and subchronic (in the last 12-72h) (30, 31) alcohol intake (**Figure 4**), without significant changes in BMI (30, 31), fasting circulating glucose, triglycerides and liver stiffness (30). Thus, the acute or subchronic alcohol consumption-induced cFGF21 elevations are more likely associated with the feedback of the liver-brain endocrine circuit and act as a liver-derived inhibitor of alcohol preference and intake (83). We also noticed that, elevation of cFGF21 tends to be higher in individuals with alcoholic liver cirrhosis than in those with nonalcoholic liver cirrhosis (**Figure 4**) (31), suggesting that chronic alcohol consumption may create a more severe “FGF21 resistance” in the liver where more cFGF21 is required to act efficiently as an alcohol preference-suppressing endocrine.

## Calorie restriction-induced weight loss and cFGF21

Obesity is associated with elevated levels of cFGF21, and the expected beneficial effects of FGF21 for improving glucose tolerance and reducing plasma glucose and triglyceride levels are attenuated or even lost in obesity, suggesting the existence of an FGF21-resistant state (84). The effects of CRIWL on the cFGF21 level have been conflicting, with many factors involved, such as the number and type of participants, type of dietary intervention, time frame following intervention type and outcome measurement, and amount of weight loss (42). In this meta-analysis, 11 studies containing 373 individuals were involved in the analysis of the association between CRIWL and cFGF21 concentration (**Table 4**). Overall, CRIWL significantly decreased the cFGF21 level (p=0.02) (**Figure 5**). Among all the factors that could affect the CRIWL-induced cFGF21 change, we found that the protein content in the diet could be a key factor with a 30% calorie from protein as a cutoff value. Most of the CRIWL-decreased cFGF21 results were obtained with relatively high dietary protein contents (≥30E% protein) (32–36), while controversial results were obtained with relatively low dietary protein contents (<30E% protein) (36–40) or with diets with unspecified protein contents (32, 41, 42).

An elegant review has recently pointed out that CR without malnutrition can reduce disease burden and prolong lifespan in humans and animals, but extreme CR can impair immunity (85). Our analysis is in line with this statement and further indicated that FGF21 could serve as a nutrition marker for monitoring the quality of CR. Numerous studies have shown that obesity and liver fat content are associated with elevated cFGF21 levels (66, 81, 84, 86); thus, a decrease in cFGF21 levels could well reflect the reduction in body fat following CRIWL without malnutrition. On the other hand, protein restriction upregulates the cFGF21 level (6, 22). Thus, a CR with protein shortage could affect the cFGF21 level in two ways: downregulation of cFGF21 *via* CR-induced fat loss and upregulation of cFGF21 *via* CR-caused protein shortage.

This could explain the different observations between the study of Crujeiras et al. (32) and that of Šrámková et al. (40) (**Figure 5**). cFGF21 significantly decreased in obese patients after one month of very low-calorie diet (VLCD, 600–800 kcal/d) with a very high protein content (~58.2E% protein) in Crujeiras et al. (32), while cFGF21 significantly increased in obese women followed 800 kcal/d VLCD with relatively lower protein content (~26.1E% protein) for 28 days in Šrámková et al. (40). The VLCD-caused protein shortage could be the dominant factor that drove this cFGF21 upregulation in Šrámková et al. (40), while the very high protein content could have compensated the VLCD-caused protein shortage, leaving the CR-induced fat loss as the dominant factor that drived this cFGF21 downregulation in Crujeiras et al. (32). We also notice that, with diets of similar or lower protein content, a moderate low calorie restriction (LCD, 1400–1850 kcal/d, 15-30E% protein) could still achieve a significant cFGF21 decrease (32), while the VLCD (800 kcal/d, ~26.1% calorie from protein) could not (40), suggesting that the increased calorie intake [LCD (32) vs. VLCD (40)] at the same time rescued the VLCD-induced protein shortage and attenuated the protein shortage-caused cFGF21 upregulation, resulting an overall decreased cFGF21 level, even with a higher overall calorie intake. Ultimately, the works of Xu et al. (36) showed that with the same level of calorie restriction (LCD, 1500-1600 kcal/d) for 3 weeks, 30E% protein could still significantly decrease cFGF21 in obese patients with lowered liver fat content and inflammation and increased lipid metabolism, whereas the 10E% protein group exhibited a significant increase in cFGF21 with deteriorated lipid metabolism, underlining the importance of protein content in the diet for successful CRIWL.

In addition, among the 10 studies included in this meta-analysis, 2 studies indicated that omega-3 fatty acids were supplemented in their VLCD (800 kcal/d) (32, 35). Fish oil is rich in omega-3 polyunsaturated fatty acids, which have been reported to have myriad health benefits, including reductions in the circulating levels of lipids, glucose and inflammatory cytokines such as TNF-α and nuclear factor-κB (87). All these factors could lead to the marked down-regulation of cFGF21 levels in the patients with nonalcoholic fatty liver disease (NAFLD) associated with hyperlipidemia after consumption of fish oil 4 g per day for 3 months (n=36) (**Figure 3**) (27). Thus, fish oil supplement could also contribute to the decrease in cFGF21 even after VLCD in these 2 CRIWL studies (32, 35).

Taken together, FGF21 could be an excellent nutrition marker for monitoring the quality of CRIWL, and its downregulation after CRIWL could serve to evaluate the fat loss effect of CRIWL, while an unaffected or upregulated cFGF21 level after CRIWL could warn of CRIWL with protein shortage or malnutrition.

## Exercise and cFGF21

Regular exercise is an effective lifestyle intervention for the prevention and treatment of many chronic metabolic diseases, including obesity, diabetes, and NAFLD (88, 89). As cFGF21 changes in response to exercise, FGF21 has been identified as an exercise-mediated hormone (45). However, there are controversial results for the impact of exercises on cFGF21, with some studies revealing that exercise increased cFGF21 levels (43, 44, 46, 47, 49, 51, 52, 54, 55), while other studies reported opposite results (50, 53, 56–61).

Exercise promotes lipolysis of adipose tissue, which is similar to fasting, and the subsequently released FFAs are utilized as an energy source through fatty acid oxidation (90). As the increased circulating FFA is also a signal leading to transcriptional upregulation of FGF21 (43, 60, 66, 71), the beneficial effects of acute exercise in improving the lipid metabolism could be partly contributed by the exercise-caused temporary FFA release, followed by the elevations of the cFGF21 levels and in turn promoting the FFA’s own consumption and fat burning (66, 90). In the case of chronic training, repeatedly enhanced lipid metabolism and fat burning by daily exercise often result in improved lipid metabolic flexibility and decreased body fat (90), and, consequently, an eventual decrease in cFGF21 levels.

With the guidance of the above theoretical predictions, we dissected our meta-analysis of the exercise-cFGF21 association according to the blood-sampling time postexercise. As expected, 10 studies containing 239 individuals showed overall significantly increased cFGF21 levels within 3 hours postexercise compared with preexercise levels (43–52) (p<0.0001) (**Figure 6**). Five of 10 studies measured cFGF21 concentrations with blood sampled at multiple time points postexercise. Among them, 4 studies showed a cFGF21 peak at 1 h postexercise (46, 49, 50, 52), one peaked at 2h postexercise (48), and all returned to baseline thereafter. This result is in line with the fact that serum FGF21 constantly undergoes proteolytic degradation with a half-life of approximately 0.5-1 h (91). Moreover, a higher intensity of acute exercise resulted in a higher cFGF21 level 1 h postexercise, which is in line with the fact that exercise with a higher intensity requires a higher availability of FFA and higher fatty acid oxidation to match the energy demands, thus resulting in a higher level of FFA-enhanced cFGF21 upregulation compared to exercise with a lower intensity (**Figure 7**).

On the other hand, 12 studies that measured cFGF21 levels in the blood sampled beyond 3 hours postexercise (48, 50, 52–61) showed an overall reduction in cFGF21 (p=0.01) (**Figure 8**). After a long postexercise recovery time, the acute exercise-induced temporary lipolysis and FFA release, as well as the FFA-induced cFGF21, can be largely eliminated. Therefore, the cFGF21 level beyond 3 hours postexercise would mainly be associated with the healthy benefits of exercise, including reduced obesity and liver fat content, decreased obesity-induced FGF21 resistance, and improved glucose and lipid metabolism. All these factors could converge to lead to a reduced cFGF21 level (66, 90) (**Figure 8**).

Taken together, it is important to differentiate the acute effect of exercise from the chronic effect of training (**Figure 9**). While acute exercise transiently increases the cFGF21 within hours postexercise, mostly with a peak at approximately 1 hour postexercise, the increase induced by a high intensity exercise displays a higher and longer ascending phase followed by a much extended descent to the baseline level compared to that induced by a relatively lower intensity exercise. Thus, the cFGF21 level at 1 h postexercise could serve as an excellent biomarker for evaluating the intensity of acute exercise, as well as the exercise-promoted fat burning. After a longer recovery time, commonly after an overnight fast (>10 hours) to exclude any temporary effects of exercise, the beneficial effects of chronic training on global glucose and lipid homeostasis could be assessed by the lowered blood FGF21 level.

Overall, future studies focusing on the relationship between exercise intervention and cFGF21 levels should particularly take into consideration the blood-sampling times postexercise. In addition, a standard protocol needs to be established, including exercise types, intensity, duration and timing of blood sampling following exercise, when studying the relationship between FGF21 and exercise.

Several limitations of our meta-analysis must be taken into consideration. First, significant heterogeneity was observed in the studies included in our meta-analysis. The discrepancies between studies may be due to differences in the number and types of participants, as well as specific interventional types and durations, also possibly influenced by the timing of the measurement of circulating FGF21 following intervention. Second, our meta-analysis only focused on smoking, physical activity, diet, alcohol consumption and weight loss. Studies on other lifestyles, such as sleep habits, were not included due to the limited number of relevant studies. Third, some original data reported in articles were transformed for meta-analysis, especially for data of median value with quartile. Those original data with skewed distributions may not be suitable for meta-analysis. Fourth, on account of the limited number of relevant clinical studies and sample sizes of individuals, which may be due to ethical restrictions, the observations of some studies were discussed in the light of known FGF21 biology summarized with cell and animal experiments. Therefore, the fact that the included articles were few in number may have introduced bias, and further studies are needed. Fifth, we only included articles published in English and also did not include unpublished articles, thus the publication bias may exist in this study.

Several modalities were applied to reduce these limitations. First, we conducted a systematic, comprehensive search in four databases. Second, we strictly stipulated the inclusion criteria, eliminating the bias caused by some potential confounding factors, and the data were independently extracted by two reviewers. Third, we conducted subgroup analyses of specific lifestyles.

## Conclusion

Serum FGF21 levels are closely associated with lifestyle interventions. They can be elevated by smoking, a hypercaloric carbohydrate-rich diet, a hypercaloric fat-rich diet, amino acid or protein restriction, excessive fructose intake and alcohol consumption, whereas fish oil supplementation and calorie restriction with sufficient protein intake significantly decreased cFGF21. Acute exercise significantly elevated cFGF21 levels within 3 hours postexercise, and stronger exercise intensity resulted in higher cFGF21 upregulation, while cFGF21 levels significantly decreased in the blood sampled 6 hours postexercise. FGF21 could serve as a potential biomarker for assessment of the effects of different lifestyle interventions. When it is used for this purpose, a standard study protocol needs to be established, especially taking into consideration the intervention types and sampling time post-intervention.

## Data availability statement

The original contributions presented in the study are included in the article/**Supplementary Material**. Further inquiries can be directed to the corresponding authors.

## Author contributions

ZQ, YZ and NY designed the study, performed the literature search and data extraction, analyzed the data, and drafted the manuscript; HN,ZY and PL performed the quality assessment of all included studies, advised on interpretation of the data and critically revised the manuscript; XW, YG, YH, JY and LR critically revised the manuscript; LZ and CZ conceived the idea for the review, performed the searches and data extraction, and critically revised the manuscript content. All authors contributed to the article and approved the submitted version.

## Funding

This work was supported by the National Key Research and Development Program of China [grant number 2020YFC2008000] to Cuntai Zhang; the National Natural Science Foundation of China [grant numbers 81873533] to Le Zhang.

## Acknowledgments

The authors would like to thank the subjects included in the studies we reviewed.

## Conflict of interest

The authors declare that the research was conducted in the absence of any commercial or financial relationships that could be construed as a potential conflict of interest.

## Publisher’s note

All claims expressed in this article are solely those of the authors and do not necessarily represent those of their affiliated organizations, or those of the publisher, the editors and the reviewers. Any product that may be evaluated in this article, or claim that may be made by its manufacturer, is not guaranteed or endorsed by the publisher.
