# Supplementary material for: Close association between lifestyle and circulating FGF21 levels: A systematic review and meta-analysis
Source: Front Endocrinol (Lausanne). 2022 Aug 25;13:984828. doi: 10.3389/fendo.2022.984828 (PMC9453313; doi:10.3389/fendo.2022.984828)
Supplement: Supplementary file 1 [file DataSheet_1.docx]

Supplementary Material

**Supplementary Method 1**

The detailed search strategy in PubMed was as follows: 1) ("fibroblast growth factor 21"[Supplementary Concept] OR "fibroblast growth factor 21"[All Fields] OR "fgf21"[All Fields]) AND ("life style"[MeSH Terms] OR ("life"[All Fields] AND "style"[All Fields]) OR "life style"[All Fields] OR "lifestyle"[All Fields] OR "lifestyles"[All Fields]); 2) ("fibroblast growth factor 21"[Supplementary Concept] OR "fibroblast growth factor 21"[All Fields] OR "fgf21"[All Fields]) AND ("smoke"[MeSH Terms] OR "smoke"[All Fields] OR "smoke s"[All Fields] OR "smoked"[All Fields] OR "smokes"[All Fields] OR "smoking"[MeSH Terms] OR "smoking"[All Fields] OR "smokings"[All Fields] OR "smoking s"[All Fields]); 3) ("fibroblast growth factor 21"[Supplementary Concept] OR "fibroblast growth factor 21"[All Fields] OR "fgf21"[All Fields]) AND ("drink"[All Fields] OR "drinking"[MeSH Terms] OR "drinking"[All Fields] OR "alcohol drinking"[MeSH Terms] OR ("alcohol"[All Fields] AND "drinking"[All Fields]) OR "alcohol drinking"[All Fields] OR "drinkings"[All Fields] OR "drinks"[All Fields]); 4) ("fibroblast growth factor 21"[Supplementary Concept] OR "fibroblast growth factor 21"[All Fields] OR "fgf21"[All Fields]) AND ("alcohol drinking"[MeSH Terms] OR ("alcohol"[All Fields] AND "drinking"[All Fields]) OR "alcohol drinking"[All Fields]); 5) ("ethanol"[MeSH Terms] OR "ethanol"[All Fields] OR "ethanols"[All Fields] OR "ethanol s"[All Fields] OR "ethanolic"[All Fields]) AND ("fibroblast growth factor 21"[Supplementary Concept] OR "fibroblast growth factor 21"[All Fields] OR "fgf21"[All Fields]); 6) ("fibroblast growth factor 21"[Supplementary Concept] OR "fibroblast growth factor 21"[All Fields] OR "fgf21"[All Fields]) AND ("exercise"[MeSH Terms] OR "exercise"[All Fields] OR "exercises"[All Fields] OR "exercise therapy"[MeSH Terms] OR ("exercise"[All Fields] AND "therapy"[All Fields]) OR "exercise therapy"[All Fields] OR "exercise s"[All Fields] OR "exercised"[All Fields] OR "exerciser"[All Fields] OR "exercisers"[All Fields] OR "exercising"[All Fields]); 7)("fibroblast growth factor 21"[Supplementary Concept] OR "fibroblast growth factor 21"[All Fields] OR "fgf21"[All Fields]) AND ("exercise"[MeSH Terms] OR "exercise"[All Fields] OR ("physical"[All Fields] AND "activity"[All Fields]) OR "physical activity"[All Fields]); 8) ("fibroblast growth factor 21"[Supplementary Concept] OR "fibroblast growth factor 21"[All Fields] OR "fgf21"[All Fields]) AND ("weight loss"[MeSH Terms] OR ("weight"[All Fields] AND "loss"[All Fields]) OR "weight loss"[All Fields]); 9) ("fibroblast growth factor 21"[Supplementary Concept] OR "fibroblast growth factor 21"[All Fields] OR "fgf21"[All Fields]) AND ("diet"[MeSH Terms] OR "diet"[All Fields]); 10) ("fibroblast growth factor 21"[Supplementary Concept] OR "fibroblast growth factor 21"[All Fields] OR "fgf21"[All Fields]) AND ("fasted"[All Fields] OR "fasting"[MeSH Terms] OR "fasting"[All Fields] OR "fastings"[All Fields] OR "fasts"[All Fields]); 11) ("fibroblast growth factor 21"[Supplementary Concept] OR "fibroblast growth factor 21"[All Fields] OR "fgf21"[All Fields]) AND ("sleep"[MeSH Terms] OR "sleep"[All Fields] OR "sleeping"[All Fields] OR "sleeps"[All Fields] OR "sleep s"[All Fields]); 12) ("fibroblast growth factor 21"[Supplementary Concept] OR "fibroblast growth factor 21"[All Fields] OR "fgf21"[All Fields]) AND ("sedentary behavior"[MeSH Terms] OR ("sedentary"[All Fields] AND "behavior"[All Fields]) OR "sedentary behavior"[All Fields]).

The same medical subject headings (MeSH) and keywords in various combinations were used in the mentioned electronic databases.

**Supplementary Table 1**

Quality assessment of the **Cohort study** by Newcastle–Ottawa Scale.

| **Included Studies** | **Selection** | | | | **Comparability** | | **Outcome** | | | **Total score** |
| --- | --- | --- | --- | --- | --- | --- | --- | --- | --- | --- |
|  | A | B | C | D | A1 | B1 | A2 | B2 | C2 |  |
| Kamizono et al 2018 | 1 | 1 | 1 | 1 | 1 | 1 | 1 | 1 | 1 | **9** |

A: Representativeness of exposed cohort. B: Representativeness of unexposed cohort. C: Ascertainment of exposure (If the exposure data was obtained from prescription database or medical record). D: Outcome was not present at start. A1: Important factor (If adjusted for the age , sex and marital status, a point was assigned.) B1: Additional factor (If adjusted for any other additional factors.) A2: Assessment of outcome. B2: Exposure Follow-up for outcomes. C2: Rate of follow-up.

**Supplementary Table 2**

Quality assessment of the **Cross-section studies** by using the Joanna Briggs Institute Checklist

| **Included Studies** | **JBI quality assessment criterias** | | | | | | | | **Total score** |
| --- | --- | --- | --- | --- | --- | --- | --- | --- | --- |
|  | ① | ② | ③ | ④ | ⑤ | ⑥ | ⑦ | ⑧ |  |
| Nakanishi et al 2015 | Y | Y | Y | Y | Y | Y | Y | Y | **8** |
| Nakanishi et al 2018 | Y | Y | Y | Y | Y | Y | Y | Y | **8** |

Y: Yes, N: No, U: Unclear, NA: Not applicable. ①: Were the criteria for inclusion in the sample clearly defined? ②: Were the study subjects and the setting described in detail? ③: Was the exposure measured in a valid and reliable way? ④: Were objective, standard criteria used for measurement of the condition? ⑤: Were confounding factors identified? ⑥: Were strategies to deal with confounding factors stated? ⑦: Were the outcomes measured in a valid and reliable way? ⑧: Was appropriate statistical analysis used?

**Supplementary Table 3**

Methodological quality of the experimental studies included (ratings on the PEDro scale)

| **Included Studies** | **Specified eligibility criteria** | **Random allocation** | **Concealed allocation** | **Baseline comparability** | **Blinding of assessors** | **Completeness of follow-up** | **Intention-to-treat-analysis** | **Between group comparisons** | **Point estimates and variability** | **Total**  **(points)** |
| --- | --- | --- | --- | --- | --- | --- | --- | --- | --- | --- |
| Banitalebi et al 2019 | 1 | 1 | 0 | 1 | 0 | 1 | 1 | 1 | 1 | **7** |
| Besse-Patin et al 2014 | 1 | 0 | 0 | 1 | 0 | 1 | 1 | 1 | 1 | **6** |
| Christodoulides et al 2009 | 1 | 1 | 0 | 1 | 0 | 1 | 1 | 0 | 1 | **6** |
| Crujeiras et al 2017 | 1 | 1 | 0 | 1 | 0 | 1 | 1 | 1 | 1 | **7** |
| Cuevas- Ramos et al 2012 | 1 | 0 | 0 | 1 | 0 | 1 | 1 | 0 | 1 | **5** |
| Desai et al 2017 | 1 | 1 | 0 | 1 | 0 | 1 | 1 | 1 | 1 | **7** |
| Dushay et al 2015 | 1 | 0 | 0 | 0 | 0 | 1 | 1 | 1 | 1 | **5** |
| Fontana et al 2016 | 1 | 1 | 0 | 1 | 0 | 1 | 1 | 1 | 1 | **7** |
| Gomez-Ambrosi et al 2017 | 1 | 0 | 0 | 1 | 0 | 1 | 1 | 1 | 1 | **6** |
| Gosby et al 2016 | 1 | 1 | 0 | 1 | 0 | 1 | 1 | 1 | 1 | **7** |
| Headland et al 2019 | 1 | 1 | 0 | 1 | 0 | 1 | 1 | 0 | 1 | **6** |
| Heilbronn et al 2013 | 1 | 0 | 0 | 1 | 0 | 1 | 1 | 0 | 1 | **5** |
| Hollstein et al 2019 | 1 | 0 | 0 | 1 | 0 | 1 | 1 | 0 | 1 | **5** |
| Johansson et al 2019 | 1 | 0 | 0 | 1 | 0 | 1 | 1 | 0 | 1 | **5** |
| Jürimäe et al 2021 | 1 | 0 | 0 | 1 | 0 | 1 | 1 | 0 | 1 | **5** |
| Kanbay et al 2021 | 1 | 1 | 0 | 1 | 0 | 1 | 1 | 1 | 1 | **7** |
| Kim et al 2013 | 1 | 0 | 0 | 1 | 0 | 1 | 1 | 1 | 1 | **6** |
| Laeger et al 2014 | 1 | 1 | 0 | 1 | 0 | 1 | 1 | 1 | 1 | **7** |
| Lanng et al 2019 | 1 | 1 | 1 | 1 | 0 | 1 | 1 | 0 | 1 | **7** |
| Lee et al 2021 | 1 | 0 | 0 | 1 | 0 | 1 | 1 | 0 | 1 | **5** |
| Lips et al 2014 | 1 | 0 | 0 | 1 | 0 | 1 | 1 | 1 | 1 | **6** |
| Lundsgaard et al 2017 | 1 | 1 | 0 | 1 | 0 | 1 | 1 | 1 | 1 | **7** |
| Mai et al 2011 | 1 | 0 | 0 | 1 | 0 | 1 | 1 | 0 | 1 | **5** |
| Melhem et al 2021 | 1 | 0 | 0 | 1 | 0 | 1 | 0 | 1 | 1 | **5** |
| Migdal et al 2018 | 1 | 0 | 0 | 1 | 0 | 1 | 1 | 0 | 1 | **5** |
| Morville et al 2018 | 1 | 1 | 0 | 1 | 0 | 1 | 1 | 1 | 1 | **7** |
| Motahari Rad et al 2020 | 1 | 1 | 0 | 1 | 0 | 1 | 1 | 1 | 1 | **7** |
| Olsen et al 2020 | 1 | 1 | 1 | 1 | 1 | 1 | 1 | 1 | 1 | **9** |
| Pérez-López et al 2021 | 1 | 1 | 0 | 1 | 0 | 1 | 0 | 1 | 1 | **6** |
| Qin et al 2015 | 1 | 1 | 1 | 1 | 1 | 1 | 1 | 1 | 1 | **9** |
| Sabaratnam et al 2018 | 1 | 0 | 0 | 1 | 0 | 1 | 1 | 1 | 1 | **6** |
| Sargeant et al 2018 | 1 | 0 | 0 | 1 | 0 | 1 | 1 | 1 | 1 | **6** |
| Shabkhiz et al 2021 | 1 | 1 | 0 | 1 | 0 | 1 | 1 | 1 | 1 | **7** |
| Slusher et al 2015 | 1 | 0 | 0 | 1 | 0 | 1 | 1 | 1 | 1 | **6** |
| Søberg et al 2018 | 1 | 0 | 0 | 1 | 0 | 1 | 1 | 0 | 1 | **5** |
| Srámková et al 2016 | 1 | 0 | 0 | 1 | 0 | 1 | 1 | 0 | 1 | **5** |
| Takahashi et al 2020 | 1 | 0 | 0 | 1 | 0 | 1 | 1 | 1 | 1 | **6** |
| Taniguchi et al 2016 (a,b) | 1 | 0 | 0 | 1 | 0 | 1 | 0 | 1 | 1 | **5** |
| Taniguchi et al 2016 (c) | 1 | 1 | 0 | 1 | 0 | 1 | 0 | 0 | 1 | **5** |
| Tanimura et al 2016 | 1 | 0 | 0 | 0 | 0 | 1 | 1 | 0 | 1 | **4** |
| Vienberg et al 2012 | 1 | 1 | 0 | 1 | 0 | 1 | 1 | 1 | 1 | **7** |
| Wagner-Skacel et al 2021 | 1 | 1 | 0 | 1 | 0 | 1 | 1 | 1 | 1 | **7** |
| Watanabe et al 2020 | 1 | 0 | 0 | 1 | 0 | 1 | 1 | 0 | 1 | **5** |
| Willis et al 2019 | 1 | 1 | 1 | 1 | 0 | 1 | 1 | 1 | 1 | **8** |
| Willis et al 2020 | 1 | 1 | 0 | 1 | 0 | 1 | 1 | 0 | 1 | **6** |
| Xu et al 2020 | 1 | 0 | 0 | 1 | 0 | 1 | 1 | 0 | 1 | **5** |
| Yang et al 2011 | 1 | 0 | 0 | 1 | 0 | 1 | 1 | 0 | 1 | **5** |

1 point awarded, 0 no point awarded

**Supplementary Figure 1.** The funnel plot of the differences of circulating fibroblast growth factor 21 concentration in individuals with or without different diets.


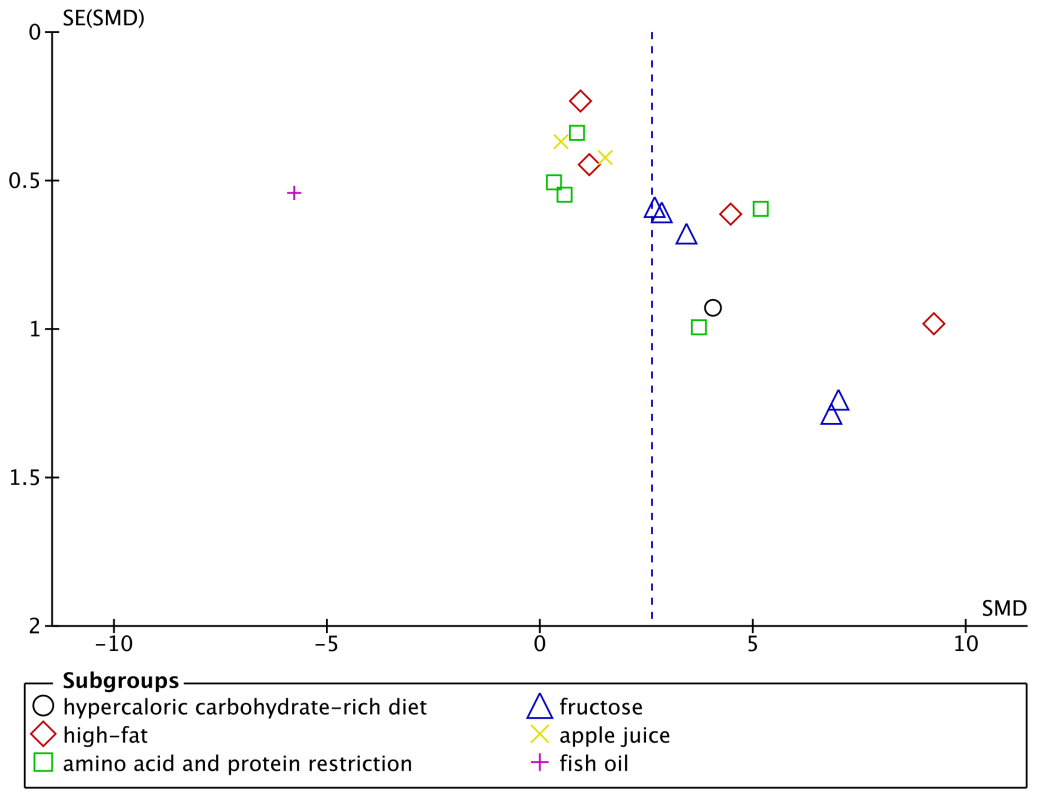


SMD: standard mean difference.

**Supplementary Figure 2.** The funnel plot of the differences of serum fibroblast growth factor 21 concentration in individuals with or without calorie restriction-induced weight loss.


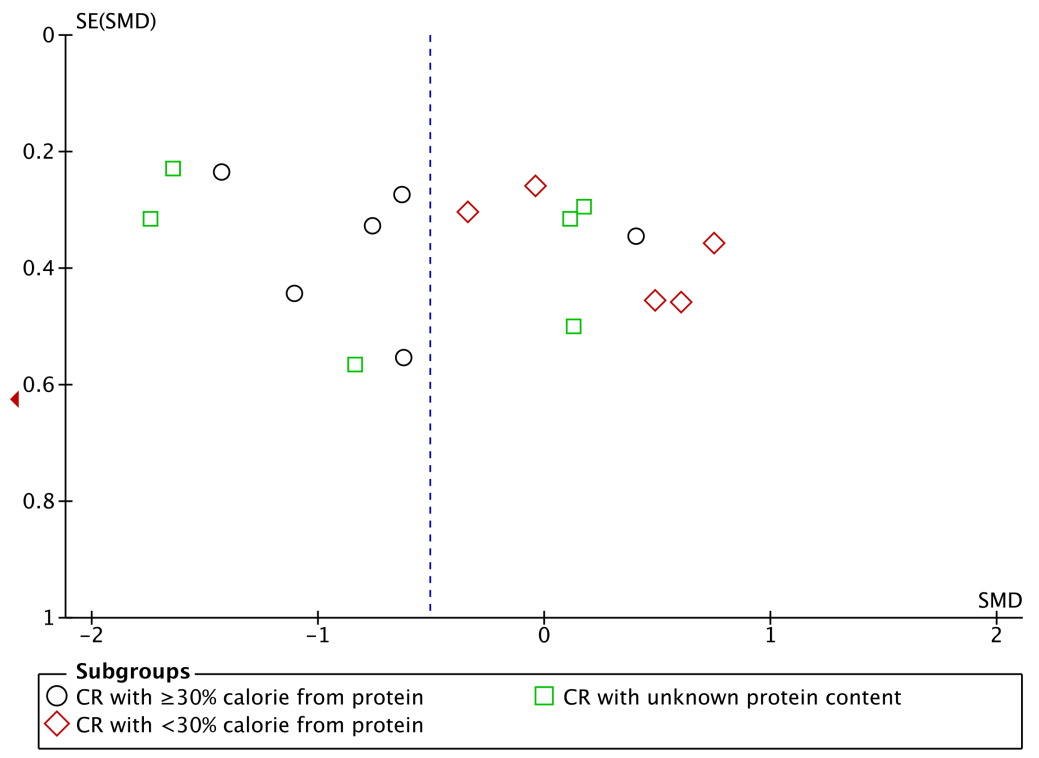


SMD: standard mean difference.

**Supplementary Figure 3.** The funnel plot of the differences of serum fibroblast growth factor 21 concentration in individuals with or without acute exercises.


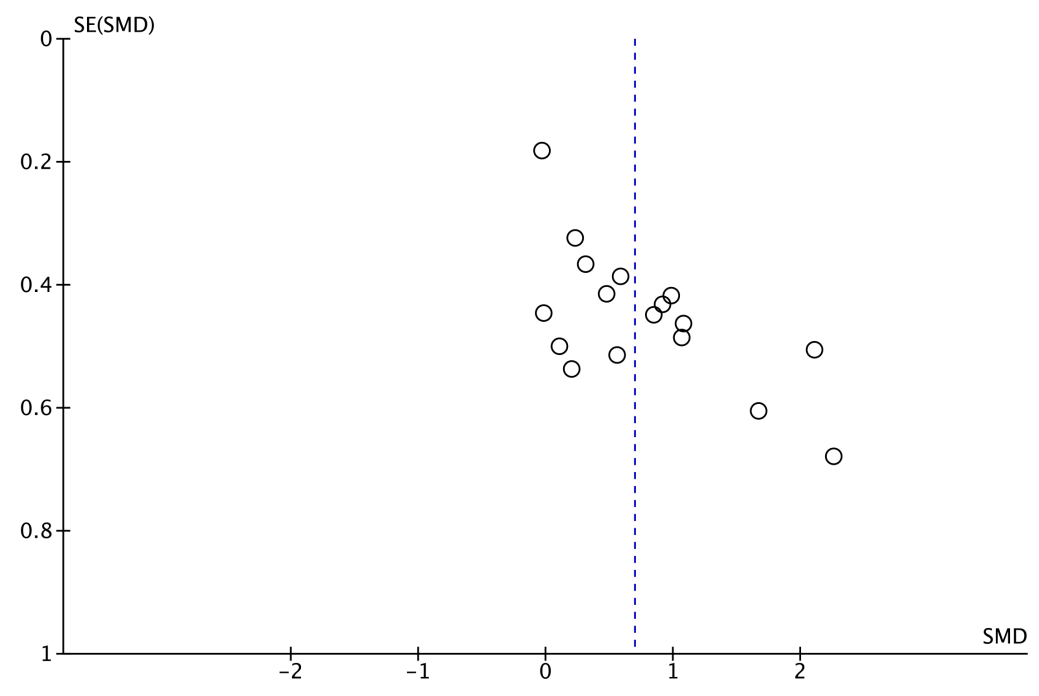


SMD: standard mean difference.

**Supplementary Figure 4.** The funnel plot of the differences of serum fibroblast growth factor 21 concentration in individuals with or without exercises measured after 6 hours.


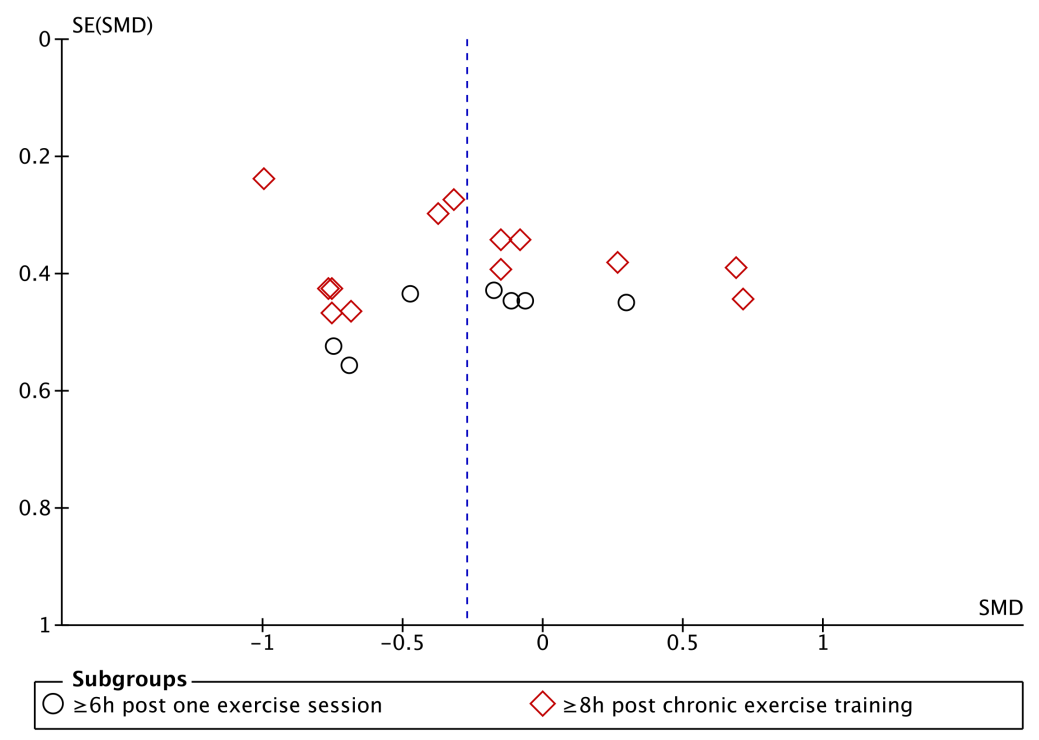


SMD: standard mean difference.
